# Prevalence and Clinical Picture of Musculoskeletal Sarcoidosis

**DOI:** 10.5812/ircmj.17918

**Published:** 2014-07-05

**Authors:** Masoumeh Salari, Zahra Rezaieyazdi

**Affiliations:** 1 Rheumatic Diseases Research Center, Ghaem Hospital, School of Medicine, Mashhad University of Medical Sciences, Mashhad, IR Iran

**Keywords:** Sarcoidosis, Arthritis, Musculoskeletal Diseases

## Abstract

**Background:**

Sarcoidosis is a multisystem disease affecting different organs with different frequency rates depending on geographical location. Musculoskeletal abnormalities includes osseous lesions (small and large bone sarcoidosis), sarcoidal arthropathy, and sarcoidal myopathy. Musculoskeletal involvement is reported in a significant number of patients.

**Objectives:**

This study aimed to determine the prevalence and clinical picture of musculoskeletal sarcoidosis in Iranian cohort of patients with sarcoidosis.

**Patients and Methods:**

We designed a descriptive cross-sectional study including 30 patients with sarcoidosis who had hospitalized in the Rheumatology Department at Ghaem Hospital, Mashhad, Iran. The patients were evaluated for musculoskeletal symptoms using history, physical examination, and paraclinical data.

**Results:**

Of the 30 studied patients, 24 were female (80%) and six were male (20%). The mean age at diagnosis was 38 years. Sarcoidal arthropathy (arthritis and periarthritis) was observed in 26 patients (86.6%). Furthermore, the initial presentation was associated with joint symptoms in 19 cases (63.3%); acute arthritis developed in 17 (65%) while bone and muscle involvements each occurred in 2 (6.6%).

**Conclusions:**

Sarcoidosis is a common disease in women aged 20 to 40 years. The most common involved joint were consecutively ankles, knees, and wrists, reaching a accumulated frequency of 86.6%; however, bone and muscle involvements were uncommon.

## 1. Background

Sarcoidosis is an inflammatory disorder characterized by noncaseating granulomas within tissues. Although the etiology of sarcoidosis remains unknown, infectious, occupational agents, and factors associated with the seasons of the year are most likely involved (1-4). It is a multisystem disease that most frequently involves lungs, lymph nodes, skin, and eyes (5). The musculoskeletal system is involved in 1% to 13% (average, 5%) of cases (1, 6). Joint involvement in sarcoidosis presents in the form of arthritis and periarthritis in up to 37% of patients (7, 8). Joints may be commonly involved in Lofgren's syndrome, which is a form of acute sarcoidosis characterized by the complex symptoms of erythema nodosum, bilateral hilar adenopathy, and polyarthritis. Arthritis has racially dependent incidence with joint symptoms seen more frequently in white females (5). The two main patterns of joint disease are acute and chronic polyarthritis with negative serology for rheumatoid factor and antinuclear antibodies.

Arthralgia, along with synovitis and periarthritis, dominates the clinical picture of sarcoidal arthropathy. Acute arthritis presents as symmetric, migratory, or additive polyarthritis that often involves the knees, ankles, wrists, elbows, and the proximal interphalangeal joints and may be associated with Lofgren's syndrome. Ankle and knee involvements are more common and typically symmetric. In patients with the acute syndrome, joint symptoms are often self-limited and are resolved within a few weeks without any permanent joint damage (9, 10). Occasionally, the isolated disease of the small joints of the hands mimics rheumatoid arthritis (5). Joint X-ray is typically unremarkable and may reveal soft tissue swelling and osteoporosis (1). Ultrasonography may show joint effusions, tenosynovitis, and subdermal edema. In a minority of patients, a chronic arthropathy develops in the early or late stages of the disease, which is characterized by a monoarticular or oligoarticular arthritis affecting the knees, ankles, and wrists; however, it does not cause joint destruction despite its chronic nature (5). Bone lesions develop in 1% to 13% of patients with sarcoidosis with an estimated average of 5% (7, 8, 11). Classic bone lesions in sarcoidosis are described in the small bones of hands and feet and are typically lytic lesions, which may lead to fracture, bone collapse, and deformity (1, 7, 12). The important diagnostic clues are often cysts in the phalanxes, which are seen in 14% of patients. The specific pattern of involvement is most evident on radiographic images, especially the images from middle and distal phalanxes. Soft tissue swelling may present with or without skin lesions on the proximal interphalangeal joints and middle phalanxes. Furthermore, pathologic fractures may occur in the rib bones or short and long tubular bones of the extremities. Fractures in the vertebral bodies may lead to spinal cord compression (11). Less common findings include distal phalangeal sclerosis with generalized dactylitis and destruction of the subchondral bone in 31% and 54% of cases, respectively, and thickening of cortical bones (11, 13). Periostitis is not common. In contrast to the more common involvement of the small bones of the extremities, the axial skeleton and long bones are seldom affected. Such lesions are rarely detected on plain X-ray images and may appear as metastatic lesions of the bone on MRI, suggesting the need for biopsy (1, 12). Nonspecific tenosynovitis, tendonitis, bursitis, and synovitis are other uncommon manifestations, which may be detected on MRI and usually require biopsy (1). Osteoporosis and soft tissue swelling may be seen on X-ray (14). Muscular sarcoidosis is reported in 50% to 80% of patients but muscle lesions were observed in less than 0.5% of cases (5, 11, 13, 15); however, symptoms occur in less than 0.5% of patients (5, 7) who may present with local pain and tenderness, muscle cramps, palpable nodules, or muscle pseudohypertrophy and contracture (8, 11, 13). Since the muscle enzymes such as creatine kinase, aldolase, and myoglobin are elevated in such patients, other diseases that are associated with elevated muscle enzymes (e.g. polymyositis) must be excluded (1, 15, 16).

## 2. Objectives

The aim of the present study was to evaluate the prevalence and clinical picture of musculoskeletal symptoms in Iranian cohort of patients with sarcoidosis who were hospitalized in the rheumatology ward of our referral university center.

## 3. Patients and Methods

This descriptive cross-sectional study was conducted on patients with sarcoidosis who were hospitalized in rheumatology ward of Ghaem Hospital, a university and referral center in northeast of Iran that has all specialty fields, over a ten-year period. Overall, from 42 patients with sarcoidosis, 12 patients were excluded due to lack of sufficient data, and 30 patients were included in this study. The definite diagnosis of sarcoidosis was made through histologic findings on biopsy in association with compatible clinical features and laboratory tests. We gathered all patients' data including clinical and paraclinical variables from their prefilled records. Therefore, we recruited patients with noncaseating granulomas in whom other etiologies such as mycobacterial, fungal, and parasitic infections as well as a history of contact with organic substances including organic bread were ruled out (8, 13). Some sarcoidosis-related syndromes such as Lofgren's syndrome do not require pathologic confirmation as long as other differential diagnoses are excluded (13). Patients without typical presentations compatible with Lofgren's syndrome, those with bilateral and symmetric hilar adenopathy but with an alternative diagnosis, or those with involvement of lung parenchyma on chest X-ray without pathologic confirmation were excluded. The patients were evaluated for sarcoidal arthropathy (arthritis and periarthritis), osseous lesions in large and small bones, and sarcoidal myopathy (myositis) by history, physical examinations, and laboratory data. This study was approved by the Ethics Committee of the Mashhad University of Medical Sciences (number 4963). The data were analyzed by SPSS (version 11.5, SPSS Inc., Chicago, IL, USA) and analyzed via descriptive statistics.

## 4. Results

Of the total number of 30 patients included in the study, 24 were female (80%) and six were male (20%). The mean age at diagnosis was 38.25 years (range, 17-56). Comorbidities amongst patients included hypertriglyceridemia in four patients (13.3%), hypertension in 2 (6.6%), diabetes mellitus in 2 (6.6%), and a history of allergies, heart attack, and obstructive lung disease each in one patient (3.3%). There were no other known rheumatic diseases in any of the patients with sarcoidosis. The most common clinical manifestations in the course of the disease were consecutively musculoskeletal in 26 cases (86.6%), dermatologic in 20 (66.6%), lower respiratory in 17 (56.6%), constitutional symptoms in 15 (50%), ophthalmic in 6 (20%), upper respiratory in 3 (10%), gastrointestinal in 2 (6.6%), involvement of the exocrine glands in 1 (3.3%), and extrathoracic lymph nodes in 1 (3.3%). Of the 30 patients, biopsies were obtained in only five patients (16.6%). In the musculoskeletal system, joint symptoms were observed in 26 patients (86.6%), acute arthritis in 17 (65.4%), periarthritis in 9 (34.6%), bone involvement in 2 (6.6%), and muscle involvement in 2 (6.6%) (Table 1). The chronic arthritis of sarcoidosis was not seen in any of the patients.

**Table 1.** Frequency of the Articular Manifestations in Patients With Sarcoidosis^a^

Findings	Patients
**Articular Manifestations**	26 (86.6)
**Acute Arthritis **	17 (65.4)
**Periarthritis**	9 (34.6)
**Chronic Arthritis**	0 (0)
**Osseous Lesions**	2 (6.6)
**Lytic Lesions**	2 (100)
**Sclerotic Lesions**	1 (50)
**Reticular Pattern**	0 (0)
**Pathologic Fracture**	0 (0)
**Myopathy Manifestations**	2 (6.6)
**Acute Myositis**	2 (100)
**Chronic Myositis**	0 (0)
**Total**	30 (100)

^a^ Data are presented as No. (%).

Lofgren's syndrome was observed in 16 patients (53.3%) including 13 women (81.2%) and three men (18.8%), among which joint symptoms and fever were reported in 15 (93.7%) and 7 (43.7%), respectively. The chronological association between joint symptoms and the development of erythema nodosum and hilar adenopathy in patients with Lofgren's syndrome are shown in Table 2. In addition, of the 18 patients (60%) with erythema nodosum, 14 were female (77.7%) and four were male (22.2%).

**Table 2.** The Association Between Lofgren Syndrome and Other Manifestations^a^

	Results
**Erythema Nodosum**	
Before the Appearance	3 (20)
At the Time of Appearance	10 (60)
After the Appearance	3 (20)
Total	16 (100)
**Hilar Adenopathy**	
Before the Detection	0 (0)
At the Time of Detection	13 (80)
After the Detection	3 (20)
Total	16 (100)

^a^ Data are presented as No. (%).

The clinical findings in patients with acute arthritis of sarcoidosis included swelling (76%), pain (70%), erythema (52%), tenderness (52%), limited range of motion (41%), pain on passive movement (23%), and warmth (17%). The frequently involved joints were the ankles (94%), knees (35%), wrists (35%), and fingers (23%) with proximal interphalangeal joints involvement in 18%, metacarpophalangeal joints in 12%, and distal interphalangeal joint in 6%. Regarding arthritis, nine out of the 17 patients with acute arthritis had oligoarthritis (53%) and the remaining eight patients (47%) had polyarthritis. All cases of polyarthritis were symmetric. Furthermore, nine patients had additive arthritis whereas there was no case of migratory arthritis. On plain X-ray, except periarticular soft tissue swelling, no other changes such as joint destruction or reduced joint space were detected. 

Bone lesions were found in only two patients (6.6%) with one patient having multiple lytic and sclerotic lesions in the fingers and toes and another with lytic lesions in both middle fingers, which were accompanied by superficial nodules. As with bone lesions, muscular involvement was observed in two patients (6.6%) presenting as symmetric muscle weakness that were more prominent in proximal than distal parts; moreover, muscle tenderness was present in one patient. Creatine phosphokinase and lactate dehydrogenase levels were elevated in both patients, but no further workup had been done to confirm myositis. Nevertheless, involvement of other organs such as lung, skin, and joints in both patients and bone lesions in one patient were accounted for the histological diagnosis of sarcoidosis. Furthermore, the muscle involvement in both cases was acute with no evidence of chronic muscle disease such as indurations or calcifications.

## 5. Discussion

Sarcoidosis is a multisystem disorder with an unknown etiology characterized by specific clinical manifestations and the histopathologic finding of noncaseating granulomas within involved tissues. The disease may present acutely or subacutely within weeks or may have an indolent course of several months. Sarcoidosis presentation is clinically significant because of the involvement of the lungs, and to a lesser extent the eyes, skin, and lymph nodes; however, joint disease, which is present in 35% of patients (1, 6), is an invaluable guide to the definite diagnosis in acute presentations. In the present study, although the disease was more common in female patients (80%), men were inflicted as well. The majority of patients (68%) aged 20 to 40 years at the time of diagnosis. In the study by Anakwenze et al. the disease was more frequent between 32 and 61 years of age with women accounting for 73% of the total number of cases (7). In general, 75% of patients are under the age of 40 years (3, 13, 17), a finding which was confirmed in the present study with similar rate (66.6%). The prevalence of joint manifestations in sarcoidosis has been reported to be between 14% and 38% (13, 17, 18). In the present study, this proportion was significantly higher (86.6%) with an even higher prevalence amongst the 16 patients with a diagnosis of Lofgren's syndrome (93.7%); however, the latter rate was 80% in the study by Moore (12). The prevalence of Lofgren's syndrome was 53.3% in our study, 4.9% in Newman et al. and 20% to 50% in Sharma et al. studies (6, 19). The higher prevalence of musculoskeletal clinical manifestations in the present study could be explained by racially dependent pevalence of arthritis (5). As in other studies, acute arthritis of sarcoidosis was more common in the ankles, knees, and wrists (7, 15). Oligoarthritis was more common than polyarthritis and the pattern of arthritis was symmetric and additive in all patients. Monoarticular arthritis was not observed in any of the patients, a similar finding to that of several other studies (5). According to the studies by Matsui and Baughman, joint disease in acute sarcoidosis may clinically manifest several weeks before the development of hilar adenopathy or in the absence of erythema nodosum (8, 13). In the present study, joint symptoms preceded erythema nodosum in 20% of patients with Lofgren's syndrome.

As mentioned earlier, regarding the skeletal system, bone lesions occurred in 6.6% of cases in the present study, while the reported prevalence is 1% to 13% (7, 8, 11, 13). The most common involved sites were the bones of the hands and feet, which had lytic lesions; however, osteosclerotic lesions were also observed. The bone lesions were accompanied by superficial skin nodules in one patient only, which is a similar finding to that of most other studies including those by Anakwenze and Moore (7, 12). According to Valencia, bone lesions are commonly asymptomatic (20). In contrast, both patients with sarcoidosis and bone lesions in the small bones of the hand and feet in our study were symptomatic. 

According to the study by Sekine et al. (15), muscles involvement was seen in 2% of patients with sarcoidosis (5, 11, 13, 15) from which less than 0.5% being symptomatic (5, 7). Such symptoms included local pain and tenderness, muscle cramps, palpable nodules, or pseudohypertrophy (8, 11, 13). In the patients in the present study, 2 (6.6%) had symptoms of myopathy that had presented as acute and symmetric muscle weakness involving mainly the proximal muscles of the limbs. Furthermore, chronic myositis was not observed in our patients.

Contrary to findings by other studies, the prevalence of acute arthritis in the present study was significantly higher (65%) due to the possible contribution of race. Since there was a significant prevalence of Lofgren’s syndrome among our patients, the role of genetic factors (e.g. HLA) and the environmental factors should be investigated more thoroughly. Therefore, it is highly recommended to contemplate the diagnosis of sarcoidosis when approaching patients with suggestive symptoms plus joint involvement, especially those of the knees, ankles, and wrists as well as muscle cramps and nodules.

### 5.1. Limitations

We evaluated only hospitalized patients; moreover, our sample size was small and the descriptive study design without any control group had negatively affected the generalization of the present study.
